# A survey dataset on China’s domestic image and media use among post-90s and post-00s cohorts

**DOI:** 10.1038/s41597-026-07591-8

**Published:** 2026-06-06

**Authors:** Boyan Zhu, Hong Cheng

**Affiliations:** 1 School of Event and Communication, Shanghai University of International Business and Economics, Shanghai, 201620 China; 2 School of Journalism and Communication, Shanghai International Studies University, Shanghai, 200083 China

**Keywords:** Communication, Sociology

## Abstract

This paper presents a survey dataset on perceptions of China’s domestic image and media use among two generational cohorts: post-90s (n = 357, collected in 2019) and post-00s (n = 1,672, collected in 2025). In this study, domestic image is defined as the perception of a country held by its own citizens and is operationalised as a multidimensional construct covering social, political, economic, cultural, and environmental domains. The dataset integrates Likert-scale measurements of national image with detailed media-use variables, enabling joint analysis of respondents’ perceptions of China’s domestic image and their media-use behaviours. The questionnaires were systematically designed and documented, with item-level transparency provided in Supplementary Materials. Technical validation includes internal consistency analysis, inter-item correlations, and both exploratory and confirmatory factor analyses. While partial structural comparability between the two cohorts is supported by overlapping measurement design, cross-cohort comparisons should be interpreted with caution due to temporal and contextual differences. This dataset provides a structured and reusable resource for research on national image, media effects, and generational differences in perception.

## Background & Summary

Understanding how individuals perceive their own country is central to research on public opinion, communication, and social cognition. In this study, national image is defined as a multidimensional and dynamic set of perceptions, evaluations, and impressions of a country held by individuals, formed through their knowledge, experiences, and exposure to information across historical and contemporary contexts^1–4^. Importantly, national image can be analytically differentiated into two interrelated fields: international image (perceptions held by foreign publics) and domestic image, which refers to how a country is perceived by its own citizens^5,6^. The latter constitutes the primary focus of this dataset and provides a foundational perspective for interpreting externally oriented national image narratives.

From a measurement perspective, domestic image is operationalised as evaluative perceptions across multiple domains, including social, political, economic, cultural, and environmental aspects. These are measured using a Likert scale. These domains are not imposed as fixed constructs but serve as a structured measurement framework that can be empirically examined using the provided data. This approach ensures conceptual clarity while maintaining flexibility for downstream analytical use.

In parallel, a substantial body of research has examined the role of mass media in shaping national image. Empirical studies show that media salience, framing, and exposure patterns influence public perceptions of countries, both directly and indirectly through agenda-setting processes^7,8^. Media use has also been shown to interact with audience predispositions, reinforcing or reshaping existing cognitive schemas^9^. While these studies provide important insights into the mechanisms linking media and perception, publicly available datasets that jointly capture individual-level perceptions of national image and detailed media-use behaviours remain limited.

This study presents a structured dataset derived from questionnaire surveys conducted among two generational cohorts in China: post-90s (born between 1 January 1990 and 31 December 1999) and post-00s (born between 1 January 2000 and 31 December 2009). Generational cohort theory suggests that values and attitudes are shaped by shared socio-economic and historical experiences during formative years^10,11^. In the Chinese context, these two cohorts have grown up under markedly different conditions in terms of economic development, digitalisation, and media environments, which may be associated with systematic differences in perception and behaviour^12–17^.

The dataset contains two subsets: one includes 357 valid observations for the post-90s collected in 2019, and the other includes 1,672 valid observations for the post-00s collected in 2025. The questionnaires include Likert-scale items capturing domestic image perceptions and detailed media-use variables, enabling joint analysis of respondents’ perceptions of China’s domestic image and their media-use behaviours within a unified data structure. Unlike many existing studies that rely on context-specific or partially documented instruments, this dataset provides (1) a systematically designed and documented questionnaire, (2) item-level transparency through Supplementary Materials, and (3) integrated measurement of national image and media use. These features enhance reproducibility and facilitate reuse across research contexts.

The dataset is suitable for descriptive and exploratory analyses. While partial alignment between the two questionnaires permits limited cross-cohort comparison at the level of constructs and measurement patterns, such comparisons should not be interpreted as causal or purely generational effects. This is due to differences in data collection periods (2019 vs. 2025), which may introduce age–period confounding effects, particularly in light of major societal disruptions such as the COVID-19 pandemic.

Consistent with many survey-based datasets, the data are based on non-probability sampling and are not nationally representative^18–21^. Accordingly, the dataset is best suited for exploratory analyses and methodological development rather than population-level inference.

## Methods

### Questionnaire Design

To design a questionnaire, the relevant principles, standards, key points, ethics and taboos should be followed^22^. We carefully considered and followed the principles of representativeness, pertinence, independence, self-consistency, completeness, readability and understandability^22^, when selecting the observation indicators of questions or variables. Meanwhile, we considered the number of items or questions, their semantic direction with both “positively-keyed” and “negatively-keyed” items, and reverse scoring of Likert scale. In order to reduce or avoid response bias wherever possible, we ensured the anonymity of all participants, facilitated diversity, and tried to make the questionnaire items concise, objective, complete, attractive and easy to answer. After designing a complete list of terms for preliminary builds of the original questionnaire, we pretested them with 43 respondents from the post-90s and 56 respondents from the post-00s (including undergraduates and graduates) to detect potential deficiencies or points of confusion, and then obtained the final version of our original questionnaire.

For the post-90s, the original questionnaire in Chinese is made up of four parts as follows: (1) A preface. (2) Socio-demographic items. (3) A five-point Likert scale, which includes 21 items. (4) A list of 4 questions pertains to media use, with each question offering four single-choice answers.

For the post-00s, the questionnaire extends the post-90s instrument, incorporating 25 Likert-scale items and 10 media-use questions. Thus, the post-00s questionnaire represents an expanded but not identical instrument, enabling partial cross-cohort comparability while preserving cohort-specific measurement features.

### Measures

The measurement instrument was developed to capture respondents’ perceptions of China’s domestic image and their media-use behaviours, with particular attention to the characteristics of the post-90s and post-00s cohorts. The questionnaire consists of a series of Likert-scale items designed to reflect multiple dimensions of national image identified in prior research, and the dimensions were informed by the “Five-sphere Integrated Plan” framework (economic, political, cultural, social, and ecological progress), which was first proposed at the 18th National Congress of the Communist Party of China in November 2012^23^, while also drawing on established multidimensional models of country image in the literature.

Drawing on established conceptualisations of country image, including cognitive and affective components^2–4,24^, as well as multidimensional frameworks^25–27^, the instrument was structured to cover several broad domains, such as social, political, economic and cultural aspects of national image. These domains were not treated as fixed theoretical constructs at the design stage; rather, they served as a guiding framework for item generation, allowing the underlying structure to be examined empirically in subsequent analyses.

All items were measured using a five-point Likert scale (1 = *Strongly Agree*, 2 = *Agree*, 3 = *Neutral*, 4 = *Disagree*, 5 = *Strongly Disagree*). The items were designed to be clear, concise and contextually relevant to contemporary Chinese society, involving economy, politics, culture, society, ecological civilization, living conditions, social environment, natural environment, wildlife protection, etc. Particular attention was paid to ensuring that the wording was appropriate for younger cohorts, who are characterised by high levels of digital media exposure and distinct socio-cultural experiences^12–15^.

The initial pool of items was developed through a combination of literature review and contextual adaptation. Specifically, items were informed by prior studies on national image, media effects, and related constructs^1,2,25–28^, while also reflecting issues salient in China’s domestic context, such as social stability, governance performance, economic development, and cultural values. Each item was reviewed and refined to ensure content relevance and linguistic clarity.

A detailed mapping of all items, including their wording, domain classification, and theoretical sources, is provided in Supplementary Table S1. No strict measurement equivalence was assumed a priori; instead, structural properties were assessed empirically, supporting cautious cross-cohort interpretation at the construct level.

### Data collection

For the post-90s, in China, using the questionnaire website “http://www.wenjuan.com”, we conducted an online survey and collected 357 valid questionnaires between 6 October 2019 and 7 November 2019 from two universities in the southeast and north of China, and several enterprises or organizations. Practical constraints in data collection resulted in a sample size of 357 respondents for the post-90s. No monetary compensation was provided to participants in the post-90s survey. All participants were China’s post-90s ranging in age from 20 to 29 during that time.

For the post-00s, data were collected through both online survey platforms and a professional questionnaire distribution company, and 1,672 valid questionnaires were collected between 3 April 2025 and 6 August 2025 from a variety of high schools, universities, enterprises, organizations, …, etc. First, we distributed the questionnaire to multiple regions in each province of China via the questionnaire website “https://wj.qq.com”, while no monetary compensation was provided to participants. Second, the company employed panel-based and network-distribution methods; however, participation incentives (if any) were managed by the survey distribution platform and were not directly controlled by the researchers, and detailed information on response rates, recruitment procedures, and incentive structures is not fully available. All participants were China’s post-00s ranging in age from 16 to 25 during that time.

Participation was voluntary and anonymous. All responses were collected through an online questionnaire system that required complete item responses prior to submission. As a result, no missing data were recorded due to enforced completion, and the sampling process should be considered non-probabilistic, and potential biases related to self-selection and platform-specific recruitment cannot be excluded. Given differences in sampling procedures, time of data collection, and social context, the two datasets should be treated as independent cross-sectional datasets rather than directly comparable samples.

By entering the website and responding to the questionnaire, all participants acknowledged that they fully understood and agreed that the data collected in the survey would be used for academic research purposes and that the dataset may be shared anonymously to ensure the objectivity and reproducibility of the research results or to be referenced by the public or researchers. The surveys did not cause harm to the human body, and did not involve sensitive personal information or commercial interests. Meanwhile, the confidentiality of the survey respondents was ensured. Ethical approval was permitted by the Ethics Committee of Shanghai University of International Business and Economics (SUIBE 2025-004). Notice that ethical approval was not required in this case due to Article 32, Paragraph (2) of the “Measures for Ethical Review of Life Sciences and Medical Research Involving Humans”, which was developed by four government agencies of China^29^.

By using software Microsoft Excel, the dataset containing the responses of post-90s and post-00s had been pre-processed, in which the answers of respondents were transformed into corresponding integers. For instance, we transformed the Location(Inside China, Outside China) in the questionnaires into Location(1, 2) in the tables or sheets of our data files (XLSX file and CSV file) as described in the following section. The rules of transformation will be described in the Readme file of our dataset.

## Data Records

The dataset with two subsets^30^, comprising ten files containing the responses of respondents of post-90s and post-00s, is available at Zenodo (https://zenodo.org/) with “10.5281/zenodo.20017889”.

For the subset of post-90s, as shown in Table 1, the five files in the dataset are: (1) Readme-China-Post90s.pdf, (2) Questionnaire-China-Post90s.pdf, (3) Questionnaire-China-Post90s-CN.pdf, (4) SurveyData-China-Post90s.csv, and (5) SurveyData-China-Post90s.xlsx.

**Table 1:** File names and types composing the subset of post-90s.

File name	File type	Description
Readme-China-Post90s.pdf	pdf	Readme file; Explanatory notes of dataset; Data transformation rules.
Questionnaire-China-Post90s.pdf	pdf	The questionnaire having a simple preface, demographic items, a Likert scale, and Media-Use items.
Questionnaire-China-Post90s-CN.pdf	pdf	The original questionnaire in Chinese
SurveyData-China-Post90s.csv	csv	Data file containing 357 valid responses
SurveyData-China-Post90s.xlsx	xlsx	Data file containing 357 valid responses.

For ease of use, the dataset contains two data files “SurveyData-China-Post90s.csv” and “SurveyData-China-Post90s.xlsx” in different formats (i.e., CSV or XLSX), which have the same schema and the same data. In this work, the original questionnaire in Chinese is the PDF file “Questionnaire-China-Post90s-CN.pdf”, which is translated into English as a PDF file “Questionnaire-China-Post90s.pdf”.

The schema of the CSV or XLSX file for the post-90s is (Gender, Age, Education, Employment, Location; LSitem1, LSitem2, …, LSitem21; MUitem1, MUitem2, MUitem3, MUitem4), in which (Gender, Age, Education, Employment, Location) are the socio-demographic items, (LSitem1, LSitem2, …, LSitem21) are the Likert-scale items, and (MUitem1, MUitem2, MUitem3, MUitem4) are the Media-Use items. Each item turns into a column of the table (or sheet) in the CSV or XLSX file, and each row in the table refers to the record/answer of a respondent.

For the subset of post-00s, as shown in Table 2, the five files in the dataset are: (1) Readme-China-Post00s.pdf, (2) Questionnaire-China-Post00s.pdf, (3) Questionnaire-China-Post00s-CN.pdf, (4) SurveyData-China-Post00s.csv, and (5) SurveyData-China-Post00s.xlsx.

**Table 2:** File names and types composing the subset of post-00s.

File name	File type	Description
Readme-China-Post00s.pdf	pdf	Readme file; Explanatory notes of dataset; Data transformation rules.
Questionnaire-China-Post00s.pdf	pdf	The questionnaire having a simple preface, demographic items, a Likert scale, and Media-Use items.
Questionnaire-China-Post00s-CN.pdf	pdf	The original questionnaire in Chinese
SurveyData-China-Post00s.csv	csv	Data file containing 1,672 valid responses
SurveyData-China-Post00s.xlsx	xlsx	Data file containing 1,672 valid responses.

For the post-00s, the original questionnaire in Chinese is the PDF file “Questionnaire-China-Post00s-CN.pdf”, which is translated into English as a PDF file “Questionnaire-China-Post00s.pdf”. The schema of the CSV or XLSX file is (Gender, Age, Education, Employment, Location; LSitem1, LSitem2, …, LSitem25; MUitem1, MUitem2, …, MUitem5.A, MUitem5.B, MUitem5.C, MUitem5.D, …, MUitem10), in which (Gender, Age, Education, Employment, Location) are the socio-demographic items, (LSitem1, LSitem2, …, LSitem25) are the Likert-scale items, and (MUitem1, MUitem2, …, MUitem5.A, MUitem5.B, MUitem5.C, MUitem5.D, …, MUitem10) are the Media-Use items. Each item turns into a column of the table (or sheet) in the CSV or XLSX file, and each row in the table refers to the record/answer of a respondent.

*Note*: The MUitem5 for the post-00s has four “Multiple-choice Answers”, while each of other questions has four “Single-choice Answers” in the list of Media-Use items of Questionnaire used for the post-00s. In the context of the post-00s, an attempt was made to design MUitem5 as a “Multiple-choice Question”.

Each subset is accompanied by a dedicated README file (Readme-China-Post90s.pdf or Readme-China-Post00s.pdf, respectively), which provides detailed documentation on variable definitions, coding schemes, and data transformation procedures. These files are intended to facilitate data reuse and ensure transparency, complementing the questionnaire instruments and tabulated metadata provided in this paper.

## Technical Validation

Technical validation includes the socio-demographic characteristics of respondents, reliability and validity analyses. The following analysis is provided to facilitate the evaluation of the validity and limitations of the data.

We utilise the software Microsoft Excel to pre-process the dataset; meanwhile, we verify whether the response data of all questionnaires is missing, even though the two online questionnaire systems require the completeness of item responses before submission. This process ensures the integrity of the dataset by confirming that all respondents have answered all questions. The data in the dataset from 357 questionnaires for post-90s and 1,672 questionnaires for post-00s have been found to be complete and valid.

**Fig. 1:**
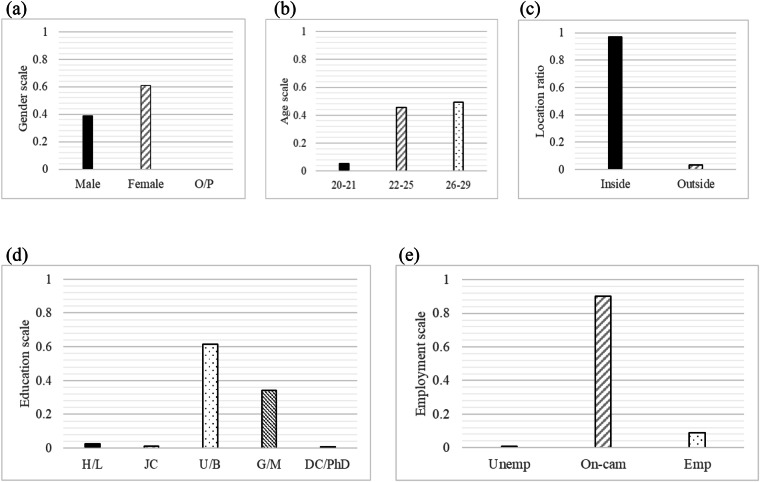
Socio-demographic characteristics of respondents for the post-90s. (**a**) Gender. (**b**) Age. (**c**) Location. (**d**) Education. (**e**) Employment.

For the post-90s, Fig. 1 illustrates the socio-demographic characteristics of 357 sample members. Gender (Fig. 1a): male (38.94%), female (61.06%), and others or prefer not to disclose (0.00%). In Fig. 1a, “O/P” means “others or prefer not to disclose”. This conforms to the general rule of questionnaire surveys, which is that female respondents usually outnumber male respondents. Age (Fig. 1b): both 20 and 21 (5.32%), between 22 and 25 (45.38%), and from 26 to 29 (49.30%). Location (Fig. 1c): Inside China (96.92%), and Outside China (3.08%). In Fig. 1c, “Inside” means “Inside China”, and “Outside” indicates “Outside China”. Education (Fig. 1d): high school or lower (2.52%), junior college (1.12%), undergraduate or bachelor’s degree (61.34%), graduate or master’s degree (34.17%), and doctoral candidate or PhD (0.84%). In Fig. 1d, “H/L” means “high school or lower”, “JC” means “junior college”, “U/B” means “undergraduate or bachelor’s degree”, “G/M” means “graduate or master’s degree”, and “DC/PhD” indicates “doctoral candidate or PhD”. Employment (Fig. 1e): unemployed (0.84%), on-campus student (90.20%), and employed (8.96%). In Fig. 1e, “Unemp” means “unemployed”, “On-cam” means “on-campus student”, and “Emp” indicates “employed”. Based on the above socio-demographic characteristics of respondents, clearly, the statistics of Age, Location, Education and Employment are consistent, with the vast majority of the respondents being on-campus undergraduate students or graduate students aged 22 to 29 inside China. The reason is that we conducted the online survey from two universities in the southeast and north of China, and several enterprises or organizations as mentioned in the Methods section above. Notice that many studies have utilised samples from college/university students^19,20^.

**Fig. 2:**
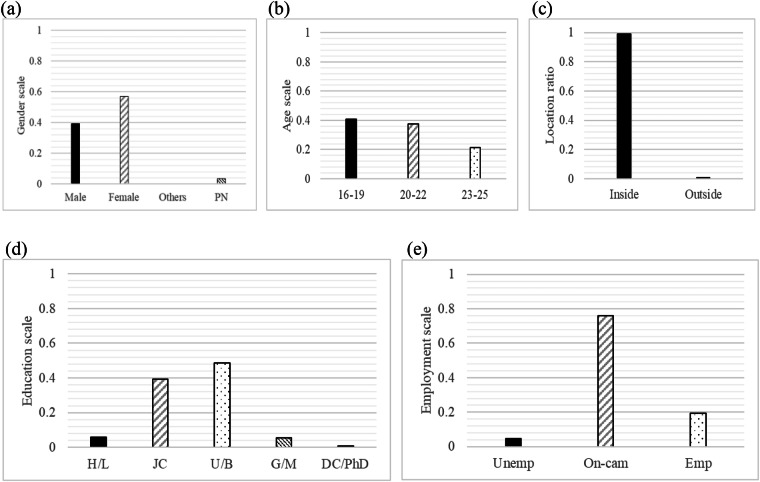
Socio-demographic characteristics of respondents for the post-00s. (**a**) Gender. (**b**) Age. (**c**) Location. (**d**) Education. (**e**) Employment.

For the post-00s, Fig. 2 demonstrates the socio-demographic characteristics of 1,672 sample members. Gender (Fig. 2a): male (39.23%), female (56.94%), others (0.42%), and prefer not to disclose (3.41%). In Fig. 2a, “PN” means “prefer not to disclose”. This conforms to the general rule of questionnaire surveys, which is that female respondents usually outnumber male respondents. Age (Fig. 2b): between 16 and 19 (40.97%), between 20 and 22 (37.68%), and from 23 to 25 (21.35%). Location (Fig. 2c): Inside China (99.16%), and Outside China (0.84%). In Fig. 2c, “Inside” means “Inside China”, and “Outside” indicates “Outside China”. Education (Fig. 2d): high school or lower (5.74%), junior college student or junior college graduate (39.17%), undergraduate or bachelor’s degree (48.56%), graduate or master’s degree (5.50%), and doctoral candidate or PhD (1.02%). In Fig. 2d, “H/L” means “high school or lower”, “JC” means “junior college student or junior college graduate”, “U/B” means “undergraduate or bachelor’s degree”, “G/M” means “graduate or master’s degree”, and “DC/PhD” indicates “doctoral candidate or PhD”. Employment (Fig. 2e): unemployed (4.72%), on-campus student (75.90%), and employed (19.38%). In Fig. 2e, “Unemp” means “unemployed”, “On-cam” means “on-campus student”, and “Emp” indicates “employed”. Based on the above socio-demographic characteristics of respondents, clearly, the statistics of Age, Location, Education and Employment are consistent. The majority of respondents are on-campus junior college students or undergraduate students aged between 16 and 25, or have just initiated their professional journeys in China^15^.

Through standard graphical user interfaces of IBM SPSS, IBM AMOS and SPSSAU, without the use of custom scripts, we measure the internal consistency, inter-item correlations, exploratory factor analysis (EFA), confirmatory factor analysis (CFA), and convergent validity (AVE, CR) of the Likert scale in our questionnaire survey. These analyses support structural validity within each cohort; however, they do not establish strict measurement invariance across cohorts. Moreover, detailed outputs are provided in the Supplementary Materials to ensure full transparency and reproducibility. In the flowing Tables 3–6, “L1, L2, …, L25” indicate “LSitem1, LSitem2, …, LSitem25”, i.e., the item numbers.

**Table 3:** Item-total statistics for the post-90s.

Item	Scale Mean if Item Deleted	Scale Variance if Item Deleted	Corrected Item-Total Correlation	Squared Multiple Correlation	Cronbach’s Alpha if Item Deleted
L1	60.266	152.645	0.023	0.517	0.866
L2	59.782	142.300	0.428	0.525	0.846
L3	59.868	143.339	0.426	0.487	0.846
L4	59.997	148.711	0.190	0.388	0.855
L5	59.894	140.213	0.554	0.388	0.841
L6	59.762	138.525	**0.652**	0.546	0.838
L7	60.011	151.550	0.091	0.296	0.859
L8	59.882	142.009	0.607	0.473	0.841
L9	59.751	140.429	0.450	0.498	0.845
L10	59.723	139.414	0.636	0.483	0.839
L11	59.737	141.110	0.502	0.602	0.843
L12	59.787	137.876	0.610	0.659	0.838
L13	59.919	140.328	0.611	0.506	0.840
L14	59.849	138.505	0.589	0.499	0.839
L15	60.146	156.580	**−0.075**	0.386	**0.867**
L16	59.824	137.668	0.620	0.544	0.838
L17	59.927	135.877	0.642	0.655	**0.837**
L18	59.950	136.874	0.644	0.632	0.837
L19	59.681	141.499	0.463	0.629	0.844
L20	59.776	141.034	0.459	0.542	0.844
L21	59.613	144.131	0.318	0.677	0.850

**Table 4:** Item-total statistics for the post-00s.

Item	Scale Mean if Item Deleted	Scale Variance if Item Deleted	Corrected Item-Total Correlation	Squared Multiple Correlation	Cronbach’s Alpha if Item Deleted
L1	52.79	214.111	0.610	0.429	0.954
L2	51.65	207.764	0.599	0.564	0.955
L3	52.89	213.217	0.669	0.505	0.954
L4	52.92	214.324	0.611	0.437	0.954
L5	53.42	218.286	0.521	0.494	0.955
L6	52.30	207.955	0.667	0.571	0.954
L7	52.56	207.212	0.744	0.630	0.953
L8	52.26	208.574	0.648	0.601	0.954
L9	52.95	213.071	0.697	0.537	0.953
L10	53.24	214.833	0.634	0.536	0.954
L11	53.18	214.922	0.652	0.587	0.954
L12	53.11	212.837	0.681	0.584	0.954
L13	53.44	217.794	0.551	0.557	0.955
L14	52.82	211.038	0.713	0.560	0.953
L15	52.87	209.768	0.739	0.573	0.953
L16	52.54	205.951	0.762	0.670	0.953
L17	52.99	211.369	0.747	0.619	0.953
L18	52.86	211.924	0.724	0.605	0.953
L19	52.84	209.612	0.733	0.649	0.953
L20	52.83	209.709	0.719	0.617	0.953
L21	53.01	211.941	0.693	0.559	0.953
L22	53.35	216.737	0.558	0.517	0.955
L23	52.86	209.370	0.707	0.598	0.953
L24	53.03	211.245	0.751	0.653	0.953
L25	52.20	205.470	0.643	0.600	0.955

**Table 5:** Inter-item correlation matrix for post-90s.

	L1	L2	L3	L4	L5	L6	L7	L8	L9	L10	L11	L12	L13	L14	L15	L16	L17	L18	L19	L20	L21
L1	1	.239**	0.036	.500**	0.092	.138**	.317**	−0.118*	−0.295**	−0.011	−0.244**	−0.179**	.138**	.166**	.418**	−0.150**	.185**	.169**	−0.380**	−0.283**	−0.495**
L2		1	.603**	.181**	.385**	.467**	.113*	.262**	.121*	.319**	0.022	0.051	.326**	.306**	.177**	.151**	.423**	.345**	0.033	0.011	−0.057
L3			1	−0.026	.325**	.477**	0.097	.332**	.184**	.413**	.161**	.195**	.270**	.255**	0.002	.217**	.333**	.334**	.148**	.127*	0.048
L4				1	0.063	0.076	.376**	0.025	−0.099	0.035	−0.1	−0.015	0.094	.200**	.318**	0.047	.240**	.245**	−0.088	−0.098	−0.163**
L5					1	.523**	0.014	.399**	.282**	.391**	.275**	.330**	.367**	.418**	0.012	.391**	.410**	.354**	.254**	.244**	.230**
L6						1	0.086	.446**	.316**	.471**	.259**	.329**	.451**	.469**	.111*	.395**	.540**	.517**	.210**	.257**	.162**
L7							1	−0.094	−0.213**	0.014	−0.177**	−0.084	0.008	0.103	.355**	−0.077	.134*	.141**	−0.035	−0.120*	−0.143**
L8								1	.462**	.491**	.500**	.515**	.436**	.370**	−0.177**	.496**	.368**	.409**	.425**	.392**	.410**
L9									1	.448**	.514**	.502**	.261**	.207**	−0.276**	.502**	.185**	.182**	.546**	.513**	.546**
L10										1	.469**	.517**	.418**	.407**	−0.165**	.500**	.434**	.393**	.388**	.375**	.302**
L11											1	.712**	.376**	.312**	−0.359**	.560**	.319**	.313**	.520**	.481**	.466**
L12												1	.508**	.423**	−0.248**	.571**	.335**	.349**	.560**	.542**	.502**
L13													1	.587**	−0.038	.398**	.482**	.479**	.285**	.302**	.163**
L14														1	−0.018	.405**	.535**	.488**	.150**	.222**	0.103
L15															1	−0.266**	0.07	.116*	−0.341**	-.261**	-.384**
L16																1	.446**	.426**	.523**	.462**	.479**
L17																	1	.744**	.186**	.211**	0.021
L18																		1	.275**	.249**	0.068
L19																			1	.612**	.687**
L20																				1	.659**
L21																					1

**Correlation is significant at the 0.01 level (2-tailed).

*Correlation is significant at the 0.05 level (2-tailed).

**Table 6:** Inter-item correlation matrix for post-00s.

	L1	L2	L3	L4	L5	L6	L7	L8	L9	L10	L11	L12	L13	L14	L15	L16	L17	L18	L19	L20	L21	L22	L23	L24	L25
L1	1	.509^**^	.475^**^	.457^**^	.327^**^	.501^**^	.465^**^	.458^**^	.394^**^	.360^**^	.398^**^	.405^**^	.293^**^	.454^**^	.439^**^	.456^**^	.439^**^	.441^**^	.437^**^	.426^**^	.402^**^	.303^**^	.485^**^	.414^**^	.453^**^
L2		1	.473^**^	.406^**^	.164^**^	.615^**^	.535^**^	.628^**^	.384^**^	.278^**^	.268^**^	.329^**^	.156^**^	.460^**^	.455^**^	.524^**^	.406^**^	.417^**^	.437^**^	.451^**^	.367^**^	.172^**^	.414^**^	.368^**^	.577^**^
L3			1	.523^**^	.443^**^	.477^**^	.515^**^	.444^**^	.557^**^	.443^**^	.441^**^	.484^**^	.408^**^	.463^**^	.516^**^	.504^**^	.504^**^	.484^**^	.483^**^	.497^**^	.464^**^	.384^**^	.435^**^	.514^**^	.390^**^
L4				1	.431^**^	.468^**^	.448^**^	.379^**^	.440^**^	.416^**^	.425^**^	.398^**^	.370^**^	.437^**^	.452^**^	.468^**^	.516^**^	.466^**^	.429^**^	.414^**^	.435^**^	.383^**^	.422^**^	.436^**^	.369^**^
L5					1	.291^**^	.372^**^	.223^**^	.420^**^	.530^**^	.548^**^	.526^**^	.579^**^	.363^**^	.368^**^	.314^**^	.411^**^	.386^**^	.343^**^	.325^**^	.406^**^	.510^**^	.349^**^	.464^**^	.157^**^
L6						1	.606^**^	.629^**^	.449^**^	.376^**^	.390^**^	.384^**^	.231^**^	.514^**^	.505^**^	.562^**^	.475^**^	.465^**^	.452^**^	.457^**^	.394^**^	.249^**^	.445^**^	.486^**^	.554^**^
L7							1	.613^**^	.570^**^	.453^**^	.465^**^	.503^**^	.349^**^	.572^**^	.550^**^	.704^**^	.555^**^	.533^**^	.530^**^	.505^**^	.489^**^	.377^**^	.525^**^	.562^**^	.539^**^
L8								1	.475^**^	.374^**^	.390^**^	.389^**^	.202^**^	.595^**^	.506^**^	.549^**^	.437^**^	.421^**^	.431^**^	.402^**^	.361^**^	.241^**^	.445^**^	.450^**^	.575^**^
L9									1	.519^**^	.520^**^	.564^**^	.459^**^	.504^**^	.544^**^	.534^**^	.531^**^	.506^**^	.527^**^	.545^**^	.521^**^	.473^**^	.462^**^	.541^**^	.394^**^
L10										1	.615^**^	.574^**^	.571^**^	.513^**^	.488^**^	.419^**^	.502^**^	.459^**^	.438^**^	.439^**^	.487^**^	.506^**^	.431^**^	.542^**^	.279^**^
L11											1	.658^**^	.545^**^	.514^**^	.506^**^	.422^**^	.483^**^	.455^**^	.450^**^	.412^**^	.472^**^	.534^**^	.481^**^	.559^**^	.335^**^
L12												1	.549^**^	.483^**^	.507^**^	.449^**^	.514^**^	.523^**^	.522^**^	.515^**^	.542^**^	.505^**^	.484^**^	.564^**^	.365^**^
L13													1	.391^**^	.425^**^	.358^**^	.465^**^	.426^**^	.414^**^	.456^**^	.506^**^	.596^**^	.386^**^	.506^**^	.157^**^
L14														1	.604^**^	.583^**^	.531^**^	.487^**^	.487^**^	.504^**^	.456^**^	.403^**^	.532^**^	.560^**^	.510^**^
L15															1	.624^**^	.588^**^	.537^**^	.588^**^	.568^**^	.544^**^	.437^**^	.533^**^	.602^**^	.510^**^
L16																1	.629^**^	.587^**^	.615^**^	.590^**^	.524^**^	.361^**^	.593^**^	.592^**^	.630^**^
L17																	1	.656^**^	.663^**^	.617^**^	.557^**^	.484^**^	.537^**^	.600^**^	.491^**^
L18																		1	.692^**^	.621^**^	.593^**^	.460^**^	.559^**^	.532^**^	.492^**^
L19																			1	.687^**^	.621^**^	.417^**^	.551^**^	.543^**^	.539^**^
L20																				1	.628^**^	.414^**^	.549^**^	.559^**^	.511^**^
L21																					1	.487^**^	.527^**^	.578^**^	.443^**^
L22																						1	.444^**^	.557^**^	.175^**^
L23																							1	.683^**^	.582^**^
L24																								1	.491^**^
L25																									1

**Correlation is significant at the 0.01 level (2-tailed).

### Internal consistency

This is achieved by employing the Cronbach’s alpha coefficient and item-level statistics. The Cronbach’s alpha coefficient is of importance in the technical validations of the data^31^ (*Note:* Kline suggested that generally, the coefficients around 0.90 are considered “excellent”, values around 0.80 are “very good”, and values around 0.70 are “adequate” ^32^).

For the post-90s (n = 357), the overall Cronbach’s alpha was 0.852 > 0.80, indicating “very good” internal consistency. As shown in Table 3, item-total statistics provide additional insight into item performance. Corrected item-total correlations range from −0.075 to 0.652. Most items exceed the commonly used threshold of 0.30, indicating acceptable contribution to the overall scale. However, several items fall below this threshold, suggesting weaker alignment with the overall construct. Cronbach’s alpha if item deleted ranges from 0.837 to 0.867, indicating that removal of individual items does not substantially improve internal consistency. These findings suggest that while the scale demonstrates acceptable reliability, certain items may benefit from refinement in future iterations.

For the post-00s (n = 1,672), Cronbach’s α = 0.955, indicating excellent internal consistency. However, it should be noted that Cronbach’s alpha is sensitive to the number of items in a scale, and high values (e.g., α > 0.90) may partly reflect item redundancy rather than increased construct validity^33–35^. Therefore, alpha values should be interpreted alongside item-total correlations and factor-analytic results.

As shown in Table 4, item-total statistics for the post-00s further confirm strong internal consistency. Corrected item-total correlations range from 0.521 to 0.762, with all items exceeding recommended thresholds. While this reflects strong coherence among items, such high values may also indicate potential redundancy. Future studies may consider scale refinement to improve parsimony.

While Cronbach’s alpha provides an estimate of internal consistency, it does not by itself establish dimensional validity. Therefore, inter-item correlations, EFA, and CFA were conducted to assess the structural properties of the instrument.

### Inter-item correlations

Inter-item correlations were examined using Pearson’s correlation coefficients. For the post-90s, in Table 5, inter-item correlations indicate moderate heterogeneity across items. While many item pairs show statistically significant positive correlations, the magnitude of correlations varies substantially, ranging from near-zero to strong associations. This pattern suggests that the questionnaire captures multiple related but distinct aspects of national image rather than a single unidimensional construct. A small number of negative or weak correlations (e.g., involving L4 and L7) further support the presence of multidimensionality. Moreover, detailed outputs of Table 5 are provided in the Supplementary Table S2 for the post-90s.

In contrast, Inter-item correlations for the post-00s in Table 6 are consistently positive and relatively strong, with most coefficients falling between 0.30 and 0.70 (p < 0.01), indicating a higher degree of internal coherence across items. These results suggest that the post-00s instrument captures a highly coherent construct, although future refinement may consider reducing redundancy to improve measurement efficiency. Moreover, detailed outputs of Table 6 are provided in the Supplementary Table S3 for the post-00s.

### Exploratory factor analysis

EFA was conducted to examine the underlying structure of the questionnaire. For the post-90s, the Kaiser–Meyer–Olkin (KMO) measure was 0.895 > 0.8, indicating meritorious sampling adequacy. Bartlett’s test of sphericity was significant (χ² = 3792.615, df = 210, p < 0.001), indicating suitability for factor analysis. Using principal component extraction, four factors with eigenvalues greater than 1 were retained, explaining 62.695% (>60%) of the total variance, as shown in Table 7. Moreover, detailed outputs of Table 7 are provided in the Supplementary Table S4.

**Table 7:** Total variance explained for post-90s.

Factor	Eigenvalue	Variance (%)	Cumulative (%)
1	7.033	33.488	33.488
2	3.632	17.297	50.786
3	1.397	6.653	57.438
4	1.104	5.257	62.695

For the post-00s, the KMO value was 0.971, indicating excellent sampling adequacy. Bartlett’s test was significant (χ² = 27,766.688, df = 300, p < 0.001). Four factors were extracted, explaining 65.981% of the total variance after rotation, as shown in Table 8. The rotated factor loadings generally exceeded 0.40 and formed a clear multidimensional structure. Moreover, detailed outputs of Table 8 are provided in the Supplementary Table S5.

**Table 8:** Total variance explained for post-00s.

Factor	Eigenvalue	Variance (%)	Cumulative (%)
1	5.023	20.094	20.094
2	4.828	19.31	39.404
3	4.623	18.492	57.896
4	2.021	8.085	65.981

### Confirmatory factor analysis

CFA was conducted to validate the factor structure identified in the EFA. Convergent validity was assessed using composite reliability (CR) and average variance extracted (AVE). To enhance interpretability and ensure consistency with the post-90s and post-00s cohorts, the item numbers were replaced with the item abbreviations, i.e., brief semantic labels of one to two words that accurately convey their meaning. This harmonisation facilitates structurally aligned cross-cohort comparison at the level of constructs, without implying generational equivalence.

For the post-90s, χ²/df = 2.844 < 3, CFI = 0.954, TLI = 0.926, IFI = 0.955, GFI = 0.931 (>0.90 for all of the four indices), and RMSEA = 0.072 < 0.08. Moreover, in Table 9, factor loadings ranged from 0.578 to 0.883. Notice that if a factor loading is found to be greater than 0.71, 0.63, or 0.55, then it is classified as being “excellent”, “very good”, or “good”, respectively^36^. Meanwhile, CR values ranged from 0.771 to 0.847 (>0.7) and AVE values ranged from 0.502 to 0.586 (>0.5). Thus, the CFA model with 16 items demonstrated good fit. Moreover, detailed outputs of Table 9 are provided in the Supplementary Table S6.

**Table 9:** The results of confirmatory factor analysis for post-90s.

Latent Variable	Item(abbrev.)	Factor Loading	CR	AVE
F1	Social harmony	0.715	0.840	0.514
	Emergency response	0.756		
	Honesty	0.795		
	Military strength	0.657		
	Foreign policy	0.649		
F2	Gov efficiency	**0.883**	0.847	0.586
	Gov integrity	0.802		
	IP respect	**0.578**		
	Overseas behaviour	0.765		
F3	Employment	0.715	0.771	0.528
	Wages	0.752		
	Infrastructure	0.713		
F4	Education	0.690	0.801	0.502
	Work ethic	0.708		
	Environment	0.753		
	Life satisfaction	0.681		

For the post-00s, χ²/df = 10.047, CFI = 0.935, TLI = 0.923, IFI = 0.935, GFI = 0.916, and RMSEA = 0.074, all of them meet the established criteria. Although χ²/df exceeded conventional threshold, this is expected in large samples (n = 1,672) due to the sensitivity of the chi-square statistic to sample size, and therefore does not necessarily indicate inadequate model fit^32,37,38^. Moreover, in Table 10, factor loadings ranged from 0.694 to 0.832, CR values ranged from 0.688 to 0.897, and AVE values ranged from 0.524 to 0.636. These results indicate acceptable convergent validity across constructs. Moreover, detailed outputs of Table 10 are provided in the Supplementary Table S7.

**Table 10:** The results of confirmatory factor analysis for post-00s.

Latent Variable	Item(abbrev.)	Factor Loading	CR	AVE
G1	Emergency response	0.703	0.883	0.557
	Foreign policy	0.701		
	Military strength	0.745		
	Environment	0.781		
	Infrastructure	0.785		
	Social harmony	0.757		
G2	Income balance	0.714	0.870	0.572
	Life satisfaction	0.737		
	Wages	0.771		
	Gov efficiency	0.796		
	Price stability	0.761		
G3	Honesty	0.800	0.897	0.636
	Media governance	0.750		
	Cultural export	0.795		
	IP respect	**0.832**		
	Overseas behaviour	0.808		
G4	Work ethic	**0.694**	0.688	0.524
	Innovation	0.753		

Moreover, discriminant validity is supported by multiple lines of evidence. For the post-90s, the multidimensional factor structure identified in the EFA and confirmed in the CFA indicates that the constructs represent distinct underlying dimensions. For the post-00s, discriminant validity is further supported by the heterotrait–monotrait ratio (HTMT), with all values below the conservative threshold of 0.85, indicating adequate separation among constructs.

To facilitate interpretation, a unified conceptual framework was applied across cohorts. Specifically, four higher-level domains were defined: National Governance & Capacity (NGC), Socioeconomic Well-being (SEW), Norms & Institutional Integrity (NII), and Human Capital & Values (HCI).

For the post-90s, the latent variables (F1, F2, F3, F4) were mapped onto these domains (NGC, NII, SEW, HCI) based on their dominant item loadings. For the post-00s, the latent variables (G1, G2, G3, G4) were aligned to the same conceptual framework and mapped onto the domains (NGC, SEW, NII, HCI).

While the factor composition exhibits partial redistribution across cohorts, the overall structure demonstrates strong conceptual convergence. This harmonised naming scheme refines earlier classifications (e.g., social, political, economic, and cultural image^39^) by adopting a more empirically grounded and internationally comparable terminology.

A comparison between Table 9 (F1–F4) and Table 10 (G1–G4) indicates a broadly consistent alignment between latent variables across the two cohorts. Specifically, F1 corresponds closely to G1, while F3 aligns with G2, supporting structural comparability. In contrast, F2 is distributed across G2 and G3, and F4 across G3 and G4, suggesting a degree of dimensional refinement in the post-00s cohort. Overall, the post-00s structure reflects a redistribution of certain dimensions (e.g., governance and socio-cultural norms), while preserving conceptual continuity with the post-90s framework. While partial structural alignment is observed, the factor composition differs across cohorts, indicating that the instruments capture related but not identical latent structures. Accordingly, cross-cohort comparison should be restricted to high-level conceptual patterns rather than direct numerical equivalence.

Taken together, these results demonstrate that both questionnaires exhibit good reliability and robust structural validity. The instruments capture a multidimensional representation of national image and are suitable for descriptive and exploratory analyses. In line with the Data Descriptor format, this validation is intended to document measurement properties rather than to test theoretical hypotheses.

### Scope and potential limitations

In this study, two related but independent questionnaire datasets were designed and collected to document respondents’ perceptions of China’s domestic image and their media-use behaviours among post-90s and post-00s cohorts. The primary purpose of this work is to provide structured and well-documented datasets rather than to conduct direct comparative analysis between cohorts. Although the two instruments share partial overlap in design, they should be treated as distinct datasets generated under different empirical contexts.

At present, there is no widely standardised survey instrument specifically developed for measuring national image, and empirical research focusing on China’s domestic image remains relatively limited. Furthermore, no directly comparable questionnaire-based datasets have been identified, which constrains the possibility of benchmarking the present data against existing studies.

An important limitation arises from the temporal gap between the two surveys. The post-90s data were collected in 2019, whereas the post-00s data were collected in 2025, a period marked by substantial societal transformations, including the COVID-19 pandemic and major shifts in the media and information environment. As a result, the two datasets should be regarded as independent cross-sectional datasets collected under distinct historical conditions. Cross-cohort comparisons are therefore exploratory and context-dependent, and should not be interpreted as evidence of purely generational differences.

In addition, both datasets were obtained using non-probability sampling strategies. The sample sizes (357 for the post-90s and 1,672 for the post-00s) represent only a small proportion of the total populations (approximately 202.1 million^39^ and 163.1 million^40^, respectively). The post-90s sample is primarily composed of university students, while the post-00s sample, although larger and more geographically diverse, also reflects platform-based recruitment and potential self-selection biases. These factors limit the generalisability of the findings, and the datasets are therefore more suitable for exploratory and methodological research within bounded contexts rather than population-level inference.

## Usage Notes

The dataset for the post-90s has been used in our previous work^39^, in which one-sample T-tests were conducted to examine respondents’ cognitive evaluations of China’s domestic image, and one-way ANOVA was applied to assess the associations between media use (in terms of time and type) and these evaluations.

Building on this approach, the dataset for the post-00s can be used to analyse respondents’ cognitive evaluations of China’s domestic image and to investigate the relationships between media use and such evaluations. In addition, the availability of both datasets enables exploratory cross-cohort comparison at descriptive and structural levels, but does not support causal inference regarding generational differences.

Future extensions of the dataset may include additional cohorts (e.g., post-10s), enabling more robust longitudinal or cross-cohort analyses under controlled temporal designs.

## Supplementary information

Supplementary Material

## Data Availability

The dataset used in this study is available on Zenodo at the following repository^30^: 10.5281/zenodo.20017889.

## Code availability

No custom code was developed or used in this study. Data preprocessing and statistical analyses were conducted using standard software packages, including Microsoft Excel, IBM SPSS, IBM AMOS, and SPSSAU. All procedures were performed through graphical user interfaces (GUI), involving menu-driven operations and dialogue-based configurations rather than scripted programming. The analytical steps (e.g., data transformation, reliability analysis, and factor analysis) are described in detail in the Methods and Technical Validation sections, allowing full reproducibility of the results using the same software tools.
