# Supplementary material for: A survey dataset on China’s domestic image and media use among post-90s and post-00s cohorts
Source: Sci Data. 2026 Jun 6;13:1087. doi: 10.1038/s41597-026-07591-8 (PMC13400608; doi:10.1038/s41597-026-07591-8)
Supplement: Supplementary file 1 — Supplementary Material [file 41597_2026_7591_MOESM1_ESM.pdf]

Supplementary Table S1. Item-level mapping, domain classification, and theoretical rationale of the Likert-scale items for post-90s and post-00s

Item-00s	Item-90s	Domain	Item description	Measurement focus	Rationale	Source
L1	L8	C	Education level per capita in China	Human capital and educational attainment	Education level reflects cultural development and long-term national capacity	[25], [26], [12]
L2	L2	E	Employment status is likely to be optimistic	Labour market expectations	Employment conditions are a core indicator of perceived economic performance	[25], [3]
L3	L5	E	Technological enterprises have independent innovation capability	Innovation capacity	Innovation reflects national competitiveness and technological development	[25], [4]
L4	L11	C	People are conscientious in their work	Work ethic	Work attitudes represent cultural values and social norms	[25], [26]
L5	L9	S	Emergency rescue measures are perfect	Crisis management capacity	Emergency response reflects governance effectiveness and social resilience	[3], [25]

Supplementary Table S1 (continued)

Item-00s	Item-90s	Domain	Item description	Measurement focus	Rationale	Source
L6	L1, L7	E	Commodity and house prices are stable	Price stability	Price stability influences perceptions of economic security and living conditions	[25]
L7	L17	P	Government office efficiency	Administrative efficiency	Efficiency reflects governance capacity and institutional performance	[25], [4]
L8	L3	E	Wage level meets daily consumption needs	Income adequacy	Income-consumption balance reflects perceived living standards	[3], [25]
L9	L4	E	State-owned enterprises are competitive	Market structure perception	The role of SOEs reflects perceptions of economic organisation and competition	[25], [4]
L10	L19	S	Social stability and harmony	Social order and cohesion	Stability and harmony are core indicators of domestic social conditions	[25], [2]

Supplementary Table S1 (continued 2)

Item-00s	Item-90s	Domain	Item description	Measurement focus	Rationale	Source
L11	L6	E	Public infrastructure is complete	Infrastructure development	Infrastructure reflects economic development and public service provision	[25], [3]
L12	L16	Eco	Attention to environment and wildlife protection	Environmental governance	Environmental protection reflects ecological awareness and sustainability efforts	[27], [24]
L13	L21	P	National defence and military strength	State power perception	Military strength contributes to perceptions of national capability and security	[2], [25]
L14	L10	S	Satisfaction with living and social environment	Subjective well-being	Overall satisfaction captures holistic perceptions of living conditions	[3], [26]
L15	~	E	Economic structure is balanced	Structural balance of the economy	Balanced development indicates sustainability and coordination across sectors	[25]

Supplementary Table S1 (continued 3)

Item-00s	Item-90s	Domain	Item description	Measurement focus	Rationale	Source
L16	L18	P	Government officials are incorruptible	Government integrity	Integrity is central to political trust and institutional legitimacy	[25], [4]
L17	L12	S	People value sincerity and honesty	Social values	Moral norms reflect perceived societal ethics	[26], [1]
L18	L13	S	Citizens are civilised and polite abroad	Social behaviour	Behaviour abroad reflects perceived national character	[1], [20]
L19	L14	P	Respect for intellectual property	Rule of law and compliance	IP protection reflects institutional quality and legal awareness	[1], [20]
L20	~	C	Competitiveness of cultural products overseas	Cultural influence and soft power	Cultural products represent national soft power and global cultural presence	[4], [24]

Supplementary Table S1 (continued 4)

Item-00s	Item-90s	Domain	Item description	Measurement focus	Rationale	Source
L21	L15	P	Government emphasis on the news industry	Media governance	Media regulation reflects state influence on information dissemination	[7], [8]
L22	L20	P	Independent and confident foreign policy	Foreign policy orientation	External political stance shapes national image domestically and internationally	[2], [1]
L23	~	S / C	Equality of educational opportunity	Social mobility and fairness	Education equality reflects both social justice and cultural development	[25], [10]
L24	~	Eco	Public healthcare safeguards citizens' health	Public health system effectiveness	Healthcare reflects social sustainability and ecological civilisation goals	[3], [25]
L25	~	E	Equalisation of wealth distribution	Income distribution fairness	Perceived inequality strongly affects economic and social evaluation	[25], [10]

Supplementary Table S1 provides a detailed description of all Likert-scale items used in this study, including their domain classification, measurement focus, theoretical rationale, and supporting literature sources.

To ensure consistency with the original datasets, item identifiers are reported in two formats. "Item-00s" (L1–L25, i.e., the shorthand for the attribute names LSitem1–LSitem25 of the Excel tables in the database) corresponds to the sequential numbering used in this table for the post-00s questionnaire, while the original variable names in the post-00s questionnaire PDF file of in the dataset are labelled as (6.1–6.25). "Item-90s" (L1–L21, i.e., the shorthand for the attribute names LSitem1–LSitem21 of the Excel tables in the database) corresponds to the subset of items used in the post-90s survey, while the original variable names in the post-90s questionnaire PDF file of the dataset are labelled as (6.1–6.21).

The ordering of items (6.1–6.25) follows the randomized sequence implemented in the survey design to minimise ordering effects and response bias, rather than domain-based grouping.

The domain classification is informed by the "Five-sphere Integrated Plan" framework (economic, political, cultural, social, and ecological progress), which was first proposed at the 18th National Congress of the Communist Party of China in November 2012[Hu], while also drawing on established multidimensional models of country image in the literature.

The "Rationale" column provides a concise explanation of how each item operationalises aspects of national image, and the "Source" column lists representative references that informed the conceptualisation of the item. These sources reflect theoretical grounding rather than direct item wording.

This table is intended to enhance transparency and reproducibility of the dataset, in line with the requirements of data descriptor publications.

Column descriptions:

Item-00s: Sequential item number (L1–L25) for Likert scale of the post-00s questionnaire used in this study.

Item-90s: Corresponding item number (L1–L21) for Likert scale in the post-90s questionnaire. Blank cells indicate items that were not included in the earlier survey.

Domain: Conceptual domain of national image measured by the item, the "Five-sphere Integrated Plan" framework, i.e., Economic (E), Political (P), Cultural (C), Social (S), and Eco-environmental (Eco) dimensions.

Item description: A brief and semantic compression of original survey question as presented to respondents.

Measurement focus: Specific aspect of perception captured by the item (e.g., governance efficiency, social trust, economic stability).

Rationale: Brief explanation of why the item is included and how it relates to national image construction.

Source: Representative references supporting the conceptual basis of the item (reference numbers correspond to the main reference list).

Supplementary Table S2. Detailed outputs of Inter-item correlation matrix for post-90s

		L1	L2	L3	L4	L5	L6	L7	L8	L9	L10	L11	L12	L13	L14	L15	L16	L17	L18	L19	L20	L21
L1	P.C	1	.239**	0.036	.500**	0.092	.138**	.317**	-.118*	-.295**	-0.011	-.244**	-.179**	.138**	.166**	.418**	-.150**	.185**	.169**	-.380**	-.283**	-.495**
	S.2		0.000	0.503	0.000	0.084	0.009	0.000	0.026	0.000	0.829	0.000	0.001	0.009	0.002	0.000	0.005	0.000	0.001	0.000	0.000	0.000
	N	357	357	357	357	357	357	357	357	357	357	357	357	357	357	357	357	357	357	357	357	357
L2	P.C	.239**	1	.603**	.181**	.385**	.467**	.113*	.262**	.121*	.319**	0.022	0.051	.326**	.306**	.177**	.151**	.423**	.345**	0.033	0.011	-0.057
	S.2	0.000		0.000	0.001	0.000	0.000	0.033	0.000	0.022	0.000	0.672	0.335	0.000	0.000	0.001	0.004	0.000	0.000	0.540	0.833	0.283
	N	357	357	357	357	357	357	357	357	357	357	357	357	357	357	357	357	357	357	357	357	357
L3	P.C	0.036	.603**	1	-0.026	.325**	.477**	0.097	.332**	.184**	.413**	.161**	.195**	.270**	.255**	0.002	.217**	.333**	.334**	.148**	.127*	0.048
	S.2	0.503	0.000		0.623	0.000	0.000	0.067	0.000	0.000	0.000	0.002	0.000	0.000	0.000	0.976	0.000	0.000	0.000	0.005	0.016	0.362
	N	357	357	357	357	357	357	357	357	357	357	357	357	357	357	357	357	357	357	357	357	357
L4	P.C	.500**	.181**	-0.026	1	0.063	0.076	.376**	0.025	-0.099	0.035	-0.100	-0.015	0.094	.200**	.318**	0.047	.240**	.245**	-0.088	-0.098	-.163**
	S.2	0.000	0.001	0.623		0.234	0.153	0.000	0.640	0.061	0.506	0.060	0.780	0.077	0.000	0.000	0.376	0.000	0.000	0.096	0.065	0.002
	N	357	357	357	357	357	357	357	357	357	357	357	357	357	357	357	357	357	357	357	357	357
L5	P.C	0.092	.385**	.325**	0.063	1	.523**	0.014	.399**	.282**	.391**	.275**	.330**	.367**	.418**	0.012	.391**	.410**	.354**	.254**	.244**	.230**
	S.2	0.084	0.000	0.000	0.234		0.000	0.786	0.000	0.000	0.000	0.000	0.000	0.000	0.000	0.822	0.000	0.000	0.000	0.000	0.000	0.000
	N	357	357	357	357	357	357	357	357	357	357	357	357	357	357	357	357	357	357	357	357	357
L6	P.C	.138**	.467**	.477**	0.076	.523**	1	0.086	.446**	.316**	.471**	.259**	.329**	.451**	.469**	.111*	.395**	.540**	.517**	.210**	.257**	.162**
	S.2	0.009	0.000	0.000	0.153	0.000		0.103	0.000	0.000	0.000	0.000	0.000	0.000	0.000	0.035	0.000	0.000	0.000	0.000	0.000	0.002
	N	357	357	357	357	357	357	357	357	357	357	357	357	357	357	357	357	357	357	357	357	357
L7	P.C	.317**	.113*	0.097	.376**	0.014	0.086	1	-0.094	-.213**	0.014	-.177**	-0.084	0.008	0.103	.355**	-0.077	.134*	.141**	-0.035	-.120*	-.143**
	S.2	0.000	0.033	0.067	0.000	0.786	0.103		0.076	0.000	0.790	0.001	0.112	0.878	0.052	0.000	0.148	0.011	0.007	0.507	0.024	0.007
	N	357	357	357	357	357	357	357	357	357	357	357	357	357	357	357	357	357	357	357	357	357
L8	P.C	-.118*	.262**	.332**	0.025	.399**	.446**	-0.094	1	.462**	.491**	.500**	.515**	.436**	.370**	-.177**	.496**	.368**	.409**	.425**	.392**	.410**
	S.2	0.026	0.000	0.000	0.640	0.000	0.000	0.076		0.000	0.000	0.000	0.000	0.000	0.000	0.001	0.000	0.000	0.000	0.000	0.000	0.000
	N	357	357	357	357	357	357	357	357	357	357	357	357	357	357	357	357	357	357	357	357	357
L9	P.C	-.295**	.121*	.184**	-0.099	.282**	.316**	-.213**	.462**	1	.448**	.514**	.502**	.261**	.207**	-.276**	.502**	.185**	.182**	.546**	.513**	.546**
	S.2	0.000	0.022	0.000	0.061	0.000	0.000	0.000	0.000		0.000	0.000	0.000	0.000	0.000	0.000	0.000	0.000	0.001	0.000	0.000	0.000
	N	357	357	357	357	357	357	357	357	357	357	357	357	357	357	357	357	357	357	357	357	357
L10	P.C	-0.011	.319**	.413**	0.035	.391**	.471**	0.014	.491**	.448**	1	.469**	.517**	.418**	.407**	-.165**	.500**	.434**	.393**	.388**	.375**	.302**
	S.2	0.829	0.000	0.000	0.506	0.000	0.000	0.790	0.000	0.000		0.000	0.000	0.000	0.000	0.002	0.000	0.000	0.000	0.000	0.000	0.000

	N	357	357	357	357	357	357	357	357	357	357	357	357	357	357	357	357	357	357	357	357	357
L11	P.C	-.244**	0.022	.161**	-0.100	.275**	.259**	-.177**	.500**	.514**	.469**	1	.712**	.376**	.312**	-.359**	.560**	.319**	.313**	.520**	.481**	.466**
	S.2	0.000	0.672	0.002	0.060	0.000	0.000	0.001	0.000	0.000	0.000		0.000	0.000	0.000	0.000	0.000	0.000	0.000	0.000	0.000	0.000
	N	357	357	357	357	357	357	357	357	357	357	357	357	357	357	357	357	357	357	357	357	357
L12	P.C	-.179**	0.051	.195**	-0.015	.330**	.329**	-0.084	.515**	.502**	.517**	.712**	1	.508**	.423**	-.248**	.571**	.335**	.349**	.560**	.542**	.502**
	S.2	0.001	0.335	0.000	0.780	0.000	0.000	0.112	0.000	0.000	0.000	0.000		0.000	0.000	0.000	0.000	0.000	0.000	0.000	0.000	0.000
	N	357	357	357	357	357	357	357	357	357	357	357	357	357	357	357	357	357	357	357	357	357
L13	P.C	.138**	.326**	.270**	0.094	.367**	.451**	0.008	.436**	.261**	.418**	.376**	.508**	1	.587**	-0.038	.398**	.482**	.479**	.285**	.302**	.163**
	S.2	0.009	0.000	0.000	0.077	0.000	0.000	0.878	0.000	0.000	0.000	0.000	0.000		0.000	0.468	0.000	0.000	0.000	0.000	0.000	0.002
	N	357	357	357	357	357	357	357	357	357	357	357	357	357	357	357	357	357	357	357	357	357
L14	P.C	.166**	.306**	.255**	.200**	.418**	.469**	0.103	.370**	.207**	.407**	.312**	.423**	.587**	1	-0.018	.405**	.535**	.488**	.150**	.222**	0.103
	S.2	0.002	0.000	0.000	0.000	0.000	0.000	0.052	0.000	0.000	0.000	0.000	0.000	0.000		0.728	0.000	0.000	0.000	0.005	0.000	0.053
	N	357	357	357	357	357	357	357	357	357	357	357	357	357	357	357	357	357	357	357	357	357
L15	P.C	.418**	.177**	0.002	.318**	0.012	.111*	.355**	-.177**	-.276**	-.165**	-.359**	-.248**	-0.038	-0.018	1	-.266**	0.070	.116*	-.341**	-.261**	-.384**
	S.2	0.000	0.001	0.976	0.000	0.822	0.035	0.000	0.001	0.000	0.002	0.000	0.000	0.468	0.728		0.000	0.189	0.028	0.000	0.000	0.000
	N	357	357	357	357	357	357	357	357	357	357	357	357	357	357	357	357	357	357	357	357	357
L16	P.C	-.150**	.151**	.217**	0.047	.391**	.395**	-0.077	.496**	.502**	.500**	.560**	.571**	.398**	.405**	-.266**	1	.446**	.426**	.523**	.462**	.479**
	S.2	0.005	0.004	0.000	0.376	0.000	0.000	0.148	0.000	0.000	0.000	0.000	0.000	0.000	0.000	0.000		0.000	0.000	0.000	0.000	0.000
	N	357	357	357	357	357	357	357	357	357	357	357	357	357	357	357	357	357	357	357	357	357
L17	P.C	.185**	.423**	.333**	.240**	.410**	.540**	.134*	.368**	.185**	.434**	.319**	.335**	.482**	.535**	0.070	.446**	1	.744**	.186**	.211**	0.021
	S.2	0.000	0.000	0.000	0.000	0.000	0.000	0.011	0.000	0.000	0.000	0.000	0.000	0.000	0.000	0.189	0.000		0.000	0.000	0.000	0.692
	N	357	357	357	357	357	357	357	357	357	357	357	357	357	357	357	357	357	357	357	357	357
L18	P.C	.169**	.345**	.334**	.245**	.354**	.517**	.141**	.409**	.182**	.393**	.313**	.349**	.479**	.488**	.116*	.426**	.744**	1	.275**	.249**	0.068
	S.2	0.001	0.000	0.000	0.000	0.000	0.000	0.007	0.000	0.001	0.000	0.000	0.000	0.000	0.000	0.028	0.000	0.000		0.000	0.000	0.200
	N	357	357	357	357	357	357	357	357	357	357	357	357	357	357	357	357	357	357	357	357	357
L19	P.C	-.380**	0.033	.148**	-0.088	.254**	.210**	-0.035	.425**	.546**	.388**	.520**	.560**	.285**	.150**	-.341**	.523**	.186**	.275**	1	.612**	.687**
	S.2	0.000	0.540	0.005	0.096	0.000	0.000	0.507	0.000	0.000	0.000	0.000	0.000	0.000	0.005	0.000	0.000	0.000	0.000		0.000	0.000
	N	357	357	357	357	357	357	357	357	357	357	357	357	357	357	357	357	357	357	357	357	357
L20	P.C	-.283**	0.011	.127*	-0.098	.244**	.257**	-.120*	.392**	.513**	.375**	.481**	.542**	.302**	.222**	-.261**	.462**	.211**	.249**	.612**	1	.659**
	S.2	0.000	0.833	0.016	0.065	0.000	0.000	0.024	0.000	0.000	0.000	0.000	0.000	0.000	0.000	0.000	0.000	0.000	0.000	0.000		0.000
	N	357	357	357	357	357	357	357	357	357	357	357	357	357	357	357	357	357	357	357	357	357
L21	P.C	-.495**	-0.057	0.048	-.163**	.230**	.162**	-.143**	.410**	.546**	.302**	.466**	.502**	.163**	0.103	-.384**	.479**	0.021	0.068	.687**	.659**	1

	S.2	0.000	0.283	0.362	0.002	0.000	0.002	0.007	0.000	0.000	0.000	0.000	0.000	0.002	0.053	0.000	0.000	0.692	0.200	0.000	0.000	
	N	357	357	357	357	357	357	357	357	357	357	357	357	357	357	357	357	357	357	357	357	357

**. Correlation is significant at the 0.01 level (2-tailed).

*. Correlation is significant at the 0.05 level (2-tailed).

P.C means Pearson Correlation

S.2 means Sig. (2-tailed)

Supplementary Table S3. Detailed outputs of Inter-item correlation matrix for post-00s

		L1	L2	L3	L4	L5	L6	L7	L8	L9	L10	L11	L12	L13	L14	L15	L16	L17	L18	L19	L20	L21	L22	L23	L24	L25
L1	P.C	1	.509**	.475**	.457**	.327**	.501**	.465**	.458**	.394**	.360**	.398**	.405**	.293**	.454**	.439**	.456**	.439**	.441**	.437**	.426**	.402**	.303**	.485**	.414**	.453**
	S.2		0.000	0.000	0.000	0.000	0.000	0.000	0.000	0.000	0.000	0.000	0.000	0.000	0.000	0.000	0.000	0.000	0.000	0.000	0.000	0.000	0.000	0.000	0.000	0.000
	N	1672	1672	1672	1672	1672	1672	1672	1672	1672	1672	1672	1672	1672	1672	1672	1672	1672	1672	1672	1672	1672	1672	1672	1672	1672
L2	P.C	.509**	1	.473**	.406**	.164**	.615**	.535**	.628**	.384**	.278**	.268**	.329**	.156**	.460**	.455**	.524**	.406**	.417**	.437**	.451**	.367**	.172**	.414**	.368**	.577**
	S.2	0.000		0.000	0.000	0.000	0.000	0.000	0.000	0.000	0.000	0.000	0.000	0.000	0.000	0.000	0.000	0.000	0.000	0.000	0.000	0.000	0.000	0.000	0.000	0.000
	N	1672	1672	1672	1672	1672	1672	1672	1672	1672	1672	1672	1672	1672	1672	1672	1672	1672	1672	1672	1672	1672	1672	1672	1672	1672
L3	P.C	.475**	.473**	1	.523**	.443**	.477**	.515**	.444**	.557**	.443**	.441**	.484**	.408**	.463**	.516**	.504**	.504**	.484**	.483**	.497**	.464**	.384**	.435**	.514**	.390**
	S.2	0.000	0.000		0.000	0.000	0.000	0.000	0.000	0.000	0.000	0.000	0.000	0.000	0.000	0.000	0.000	0.000	0.000	0.000	0.000	0.000	0.000	0.000	0.000	0.000
	N	1672	1672	1672	1672	1672	1672	1672	1672	1672	1672	1672	1672	1672	1672	1672	1672	1672	1672	1672	1672	1672	1672	1672	1672	1672
L4	P.C	.457**	.406**	.523**	1	.431**	.468**	.448**	.379**	.440**	.416**	.425**	.398**	.370**	.437**	.452**	.468**	.516**	.466**	.429**	.414**	.435**	.383**	.422**	.436**	.369**
	S.2	0.000	0.000	0.000		0.000	0.000	0.000	0.000	0.000	0.000	0.000	0.000	0.000	0.000	0.000	0.000	0.000	0.000	0.000	0.000	0.000	0.000	0.000	0.000	0.000
	N	1672	1672	1672	1672	1672	1672	1672	1672	1672	1672	1672	1672	1672	1672	1672	1672	1672	1672	1672	1672	1672	1672	1672	1672	1672
L5	P.C	.327**	.164**	.443**	.431**	1	.291**	.372**	.223**	.420**	.530**	.548**	.526**	.579**	.363**	.368**	.314**	.411**	.386**	.343**	.325**	.406**	.510**	.349**	.464**	.157**
	S.2	0.000	0.000	0.000	0.000		0.000	0.000	0.000	0.000	0.000	0.000	0.000	0.000	0.000	0.000	0.000	0.000	0.000	0.000	0.000	0.000	0.000	0.000	0.000	0.000
	N	1672	1672	1672	1672	1672	1672	1672	1672	1672	1672	1672	1672	1672	1672	1672	1672	1672	1672	1672	1672	1672	1672	1672	1672	1672
L6	P.C	.501**	.615**	.477**	.468**	.291**	1	.606**	.629**	.449**	.376**	.390**	.384**	.231**	.514**	.505**	.562**	.475**	.465**	.452**	.457**	.394**	.249**	.445**	.486**	.554**
	S.2	0.000	0.000	0.000	0.000	0.000		0.000	0.000	0.000	0.000	0.000	0.000	0.000	0.000	0.000	0.000	0.000	0.000	0.000	0.000	0.000	0.000	0.000	0.000	0.000
	N	1672	1672	1672	1672	1672	1672	1672	1672	1672	1672	1672	1672	1672	1672	1672	1672	1672	1672	1672	1672	1672	1672	1672	1672	1672
L7	P.C	.465**	.535**	.515**	.448**	.372**	.606**	1	.613**	.570**	.453**	.465**	.503**	.349**	.572**	.550**	.704**	.555**	.533**	.530**	.505**	.489**	.377**	.525**	.562**	.539**
	S.2	0.000	0.000	0.000	0.000	0.000	0.000		0.000	0.000	0.000	0.000	0.000	0.000	0.000	0.000	0.000	0.000	0.000	0.000	0.000	0.000	0.000	0.000	0.000	0.000
	N	1672	1672	1672	1672	1672	1672	1672	1672	1672	1672	1672	1672	1672	1672	1672	1672	1672	1672	1672	1672	1672	1672	1672	1672	1672
L8	P.C	.458**	.628**	.444**	.379**	.223**	.629**	.613**	1	.475**	.374**	.390**	.389**	.202**	.595**	.506**	.549**	.437**	.421**	.431**	.402**	.361**	.241**	.445**	.450**	.575**
	S.2	0.000	0.000	0.000	0.000	0.000	0.000	0.000		0.000	0.000	0.000	0.000	0.000	0.000	0.000	0.000	0.000	0.000	0.000	0.000	0.000	0.000	0.000	0.000	0.000
	N	1672	1672	1672	1672	1672	1672	1672	1672	1672	1672	1672	1672	1672	1672	1672	1672	1672	1672	1672	1672	1672	1672	1672	1672	1672

L9	P.C	.394**	.384**	.557**	.440**	.420**	.449**	.570**	.475**	1	.519**	.520**	.564**	.459**	.504**	.544**	.534**	.531**	.506**	.527**	.545**	.521**	.473**	.462**	.541**	.394**
	S.2	0.000	0.000	0.000	0.000	0.000	0.000	0.000	0.000		0.000	0.000	0.000	0.000	0.000	0.000	0.000	0.000	0.000	0.000	0.000	0.000	0.000	0.000	0.000	0.000
	N	1672	1672	1672	1672	1672	1672	1672	1672	1672	1672	1672	1672	1672	1672	1672	1672	1672	1672	1672	1672	1672	1672	1672	1672	1672
L10	P.C	.360**	.278**	.443**	.416**	.530**	.376**	.453**	.374**	.519**	1	.615**	.574**	.571**	.513**	.488**	.419**	.502**	.459**	.438**	.439**	.487**	.506**	.431**	.542**	.279**
	S.2	0.000	0.000	0.000	0.000	0.000	0.000	0.000	0.000	0.000		0.000	0.000	0.000	0.000	0.000	0.000	0.000	0.000	0.000	0.000	0.000	0.000	0.000	0.000	0.000
	N	1672	1672	1672	1672	1672	1672	1672	1672	1672	1672	1672	1672	1672	1672	1672	1672	1672	1672	1672	1672	1672	1672	1672	1672	1672
L11	P.C	.398**	.268**	.441**	.425**	.548**	.390**	.465**	.390**	.520**	.615**	1	.658**	.545**	.514**	.506**	.422**	.483**	.455**	.450**	.412**	.472**	.534**	.481**	.559**	.335**
	S.2	0.000	0.000	0.000	0.000	0.000	0.000	0.000	0.000	0.000	0.000		0.000	0.000	0.000	0.000	0.000	0.000	0.000	0.000	0.000	0.000	0.000	0.000	0.000	0.000
	N	1672	1672	1672	1672	1672	1672	1672	1672	1672	1672	1672	1672	1672	1672	1672	1672	1672	1672	1672	1672	1672	1672	1672	1672	1672
L12	P.C	.405**	.329**	.484**	.398**	.526**	.384**	.503**	.389**	.564**	.574**	.658**	1	.549**	.483**	.507**	.449**	.514**	.523**	.522**	.515**	.542**	.505**	.484**	.564**	.365**
	S.2	0.000	0.000	0.000	0.000	0.000	0.000	0.000	0.000	0.000	0.000	0.000		0.000	0.000	0.000	0.000	0.000	0.000	0.000	0.000	0.000	0.000	0.000	0.000	0.000
	N	1672	1672	1672	1672	1672	1672	1672	1672	1672	1672	1672	1672	1672	1672	1672	1672	1672	1672	1672	1672	1672	1672	1672	1672	1672
L13	P.C	.293**	.156**	.408**	.370**	.579**	.231**	.349**	.202**	.459**	.571**	.545**	.549**	1	.391**	.425**	.358**	.465**	.426**	.414**	.456**	.506**	.596**	.386**	.506**	.157**
	S.2	0.000	0.000	0.000	0.000	0.000	0.000	0.000	0.000	0.000	0.000	0.000	0.000		0.000	0.000	0.000	0.000	0.000	0.000	0.000	0.000	0.000	0.000	0.000	0.000
	N	1672	1672	1672	1672	1672	1672	1672	1672	1672	1672	1672	1672	1672	1672	1672	1672	1672	1672	1672	1672	1672	1672	1672	1672	1672
L14	P.C	.454**	.460**	.463**	.437**	.363**	.514**	.572**	.595**	.504**	.513**	.514**	.483**	.391**	1	.604**	.583**	.531**	.487**	.487**	.504**	.456**	.403**	.532**	.560**	.510**
	S.2	0.000	0.000	0.000	0.000	0.000	0.000	0.000	0.000	0.000	0.000	0.000	0.000	0.000		0.000	0.000	0.000	0.000	0.000	0.000	0.000	0.000	0.000	0.000	0.000
	N	1672	1672	1672	1672	1672	1672	1672	1672	1672	1672	1672	1672	1672	1672	1672	1672	1672	1672	1672	1672	1672	1672	1672	1672	1672
L15	P.C	.439**	.455**	.516**	.452**	.368**	.505**	.550**	.506**	.544**	.488**	.506**	.507**	.425**	.604**	1	.624**	.588**	.537**	.588**	.568**	.544**	.437**	.533**	.602**	.510**
	S.2	0.000	0.000	0.000	0.000	0.000	0.000	0.000	0.000	0.000	0.000	0.000	0.000	0.000	0.000		0.000	0.000	0.000	0.000	0.000	0.000	0.000	0.000	0.000	0.000
	N	1672	1672	1672	1672	1672	1672	1672	1672	1672	1672	1672	1672	1672	1672	1672	1672	1672	1672	1672	1672	1672	1672	1672	1672	1672
L16	P.C	.456**	.524**	.504**	.468**	.314**	.562**	.704**	.549**	.534**	.419**	.422**	.449**	.358**	.583**	.624**	1	.629**	.587**	.615**	.590**	.524**	.361**	.593**	.592**	.630**
	S.2	0.000	0.000	0.000	0.000	0.000	0.000	0.000	0.000	0.000	0.000	0.000	0.000	0.000	0.000	0.000		0.000	0.000	0.000	0.000	0.000	0.000	0.000	0.000	0.000
	N	1672	1672	1672	1672	1672	1672	1672	1672	1672	1672	1672	1672	1672	1672	1672	1672	1672	1672	1672	1672	1672	1672	1672	1672	1672
L17	P.C	.439**	.406**	.504**	.516**	.411**	.475**	.555**	.437**	.531**	.502**	.483**	.514**	.465**	.531**	.588**	.629**	1	.656**	.663**	.617**	.557**	.484**	.537**	.600**	.491**
	S.2	0.000	0.000	0.000	0.000	0.000	0.000	0.000	0.000	0.000	0.000	0.000	0.000	0.000	0.000	0.000	0.000		0.000	0.000	0.000	0.000	0.000	0.000	0.000	0.000

	N	1672	1672	1672	1672	1672	1672	1672	1672	1672	1672	1672	1672	1672	1672	1672	1672	1672	1672	1672	1672	1672	1672	1672	1672	1672
L18	P.C	.441**	.417**	.484**	.466**	.386**	.465**	.533**	.421**	.506**	.459**	.455**	.523**	.426**	.487**	.537**	.587**	.656**	1	.692**	.621**	.593**	.460**	.559**	.532**	.492**
	S.2	0.000	0.000	0.000	0.000	0.000	0.000	0.000	0.000	0.000	0.000	0.000	0.000	0.000	0.000	0.000	0.000	0.000		0.000	0.000	0.000	0.000	0.000	0.000	0.000
	N	1672	1672	1672	1672	1672	1672	1672	1672	1672	1672	1672	1672	1672	1672	1672	1672	1672	1672	1672	1672	1672	1672	1672	1672	1672
L19	P.C	.437**	.437**	.483**	.429**	.343**	.452**	.530**	.431**	.527**	.438**	.450**	.522**	.414**	.487**	.588**	.615**	.663**	.692**	1	.687**	.621**	.417**	.551**	.543**	.539**
	S.2	0.000	0.000	0.000	0.000	0.000	0.000	0.000	0.000	0.000	0.000	0.000	0.000	0.000	0.000	0.000	0.000	0.000	0.000		0.000	0.000	0.000	0.000	0.000	0.000
	N	1672	1672	1672	1672	1672	1672	1672	1672	1672	1672	1672	1672	1672	1672	1672	1672	1672	1672	1672	1672	1672	1672	1672	1672	1672
L20	P.C	.426**	.451**	.497**	.414**	.325**	.457**	.505**	.402**	.545**	.439**	.412**	.515**	.456**	.504**	.568**	.590**	.617**	.621**	.687**	1	.628**	.414**	.549**	.559**	.511**
	S.2	0.000	0.000	0.000	0.000	0.000	0.000	0.000	0.000	0.000	0.000	0.000	0.000	0.000	0.000	0.000	0.000	0.000	0.000	0.000		0.000	0.000	0.000	0.000	0.000
	N	1672	1672	1672	1672	1672	1672	1672	1672	1672	1672	1672	1672	1672	1672	1672	1672	1672	1672	1672	1672	1672	1672	1672	1672	1672
L21	P.C	.402**	.367**	.464**	.435**	.406**	.394**	.489**	.361**	.521**	.487**	.472**	.542**	.506**	.456**	.544**	.524**	.557**	.593**	.621**	.628**	1	.487**	.527**	.578**	.443**
	S.2	0.000	0.000	0.000	0.000	0.000	0.000	0.000	0.000	0.000	0.000	0.000	0.000	0.000	0.000	0.000	0.000	0.000	0.000	0.000	0.000		0.000	0.000	0.000	0.000
	N	1672	1672	1672	1672	1672	1672	1672	1672	1672	1672	1672	1672	1672	1672	1672	1672	1672	1672	1672	1672	1672	1672	1672	1672	1672
L22	P.C	.303**	.172**	.384**	.383**	.510**	.249**	.377**	.241**	.473**	.506**	.534**	.505**	.596**	.403**	.437**	.361**	.484**	.460**	.417**	.414**	.487**	1	.444**	.557**	.175**
	S.2	0.000	0.000	0.000	0.000	0.000	0.000	0.000	0.000	0.000	0.000	0.000	0.000	0.000	0.000	0.000	0.000	0.000	0.000	0.000	0.000	0.000		0.000	0.000	0.000
	N	1672	1672	1672	1672	1672	1672	1672	1672	1672	1672	1672	1672	1672	1672	1672	1672	1672	1672	1672	1672	1672	1672	1672	1672	1672
L23	P.C	.485**	.414**	.435**	.422**	.349**	.445**	.525**	.445**	.462**	.431**	.481**	.484**	.386**	.532**	.533**	.593**	.537**	.559**	.551**	.549**	.527**	.444**	1	.683**	.582**
	S.2	0.000	0.000	0.000	0.000	0.000	0.000	0.000	0.000	0.000	0.000	0.000	0.000	0.000	0.000	0.000	0.000	0.000	0.000	0.000	0.000	0.000	0.000		0.000	0.000
	N	1672	1672	1672	1672	1672	1672	1672	1672	1672	1672	1672	1672	1672	1672	1672	1672	1672	1672	1672	1672	1672	1672	1672	1672	1672
L24	P.C	.414**	.368**	.514**	.436**	.464**	.486**	.562**	.450**	.541**	.542**	.559**	.564**	.506**	.560**	.602**	.592**	.600**	.532**	.543**	.559**	.578**	.557**	.683**	1	.491**
	S.2	0.000	0.000	0.000	0.000	0.000	0.000	0.000	0.000	0.000	0.000	0.000	0.000	0.000	0.000	0.000	0.000	0.000	0.000	0.000	0.000	0.000	0.000	0.000		0.000
	N	1672	1672	1672	1672	1672	1672	1672	1672	1672	1672	1672	1672	1672	1672	1672	1672	1672	1672	1672	1672	1672	1672	1672	1672	1672
L25	P.C	.453**	.577**	.390**	.369**	.157**	.554**	.539**	.575**	.394**	.279**	.335**	.365**	.157**	.510**	.510**	.630**	.491**	.492**	.539**	.511**	.443**	.175**	.582**	.491**	1
	S.2	0.000	0.000	0.000	0.000	0.000	0.000	0.000	0.000	0.000	0.000	0.000	0.000	0.000	0.000	0.000	0.000	0.000	0.000	0.000	0.000	0.000	0.000	0.000	0.000	
	N	1672	1672	1672	1672	1672	1672	1672	1672	1672	1672	1672	1672	1672	1672	1672	1672	1672	1672	1672	1672	1672	1672	1672	1672	1672

**. Correlation is significant at the 0.01 level (2-tailed).
P.C means Pearson Correlation
S.2 means Sig. (2-tailed)

Supplementary Table S4. Exploratory factor analysis (EFA) and some statistics for the post-90s cohort using IBM SPSS

Table S4.1 Summary Item Statistics for the post-90s cohort

		N	%
Cases	Valid	357	100.0
	Excluded[a]	0	0.0
	Total	357	100.0

a. Listwise deletion based on all variables in the procedure.

Table S4.2 Reliability Statistics for the post-90s cohort

Cronbach's Alpha	N of Items
0.852	21

Table S4.3 KMO and Bartlett's Test for the post-90s cohort

Kaiser-Meyer-Olkin Measure of Sampling Adequacy.		0.895
Bartlett's Test of Sphericity	Approx. Chi-Square	3792.615
	df	210
	Sig.	0.000

Table S4.4 Total Variance Explained for the post-90s cohort

Component	Initial Eigenvalues			Extraction Sums of Squared Loadings		
	Total	% of Variance	Cumulative %	Total	% of Variance	Cumulative %
1	7.033	33.488	33.488	7.033	33.488	33.488
2	3.632	17.297	50.786	3.632	17.297	50.786
3	1.397	6.653	57.438	1.397	6.653	57.438
4	1.104	5.257	62.695	1.104	5.257	62.695
5	0.783	3.726	66.421			
6	0.759	3.616	70.037			
7	0.704	3.351	73.388			
8	0.642	3.055	76.443			
9	0.607	2.893	79.336			
10	0.535	2.547	81.883			
11	0.498	2.371	84.253			
12	0.467	2.222	86.475			
13	0.421	2.005	88.480			
14	0.406	1.933	90.413			
15	0.392	1.868	92.281			
16	0.343	1.633	93.915			
17	0.327	1.558	95.473			
18	0.285	1.355	96.828			
19	0.237	1.129	97.957			
20	0.226	1.074	99.032			
21	0.203	0.968	100.000			

Extraction Method: Principal Component Analysis.

Table S4.5 Component Matrix[a] for the post-90s cohort

	Component			
	1	2	3	4
L1	-0.147	0.737	0.228	-0.087
L2	0.373	0.550	-0.474	0.217
L3	0.454	0.330	-0.577	0.263
L4	0.027	0.545	0.551	0.187
L5	0.584	0.255	-0.182	0.057
L6	0.641	0.402	-0.239	0.078
L7	-0.061	0.476	0.383	0.524
L8	0.724	-0.019	-0.071	-0.004
L9	0.651	-0.342	-0.046	0.155
L10	0.718	0.090	-0.099	0.037
L11	0.710	-0.300	0.141	-0.200
L12	0.767	-0.204	0.231	-0.121
L13	0.647	0.268	0.076	-0.315
L14	0.599	0.376	0.126	-0.325
L15	-0.255	0.605	0.136	0.274
L16	0.757	-0.107	0.148	-0.048
L17	0.618	0.491	0.063	-0.187
L18	0.615	0.448	0.136	-0.112
L19	0.661	-0.416	0.172	0.313
L20	0.642	-0.368	0.172	0.212
L21	0.574	-0.574	0.115	0.328

Extraction Method: Principal Component Analysis.

a. 4 components extracted.

Table S4.6 Cronbach's alpha coefficients of four latent variables for the post-90s cohort

	Cronbach's alpha	N of Items
F1	.867	5
F2	.832	4
F3	.762	3
F4	.799	4

Supplementary Table S5. Exploratory factor analysis (EFA) and some statistics for the post-00s cohort using SPSSAU.

Table S5.1 Summary Item Statistics for the post-00s cohort

		N	%
Cases	Valid	1672	100.0
	Excluded[a]	0	0.0
	Total	1672	100.0

a. Listwise deletion based on all variables in the procedure.

Table S5.2 Reliability Statistics for the post-00s cohort

Cronbach's Alpha	N of Items
0.955	25

Table S5.3 KMO and Bartlett's Test for the post-00s cohort

Kaiser-Meyer-Olkin Measure of Sampling Adequacy.		0.971
Bartlett's Test of Sphericity	Approx. Chi-Square	27766.688
	df	300
	Sig.	0.000

Table S5.4 Total Variance Explained for the post-00s cohort

Component	Eigenvalues			Pre-rotation variance explained rate			Post-rotation variance explained rate		
	Eigenvalues	% of Variance	Cumulative %	Eigenvalues	% of Variance	Cumulative %	Eigenvalues	% of Variance	Cumulative %
1	12.423	49.691	49.691	12.423	49.691	49.691	**5.023**	**20.094**	**20.094**
2	2.131	8.523	58.214	2.131	8.523	58.214	**4.828**	**19.31**	**39.404**
3	1.113	4.453	62.667	1.113	4.453	62.667	**4.623**	**18.492**	**57.896**
4	0.829	3.315	65.982	0.829	3.315	65.982	**2.021**	**8.085**	**65.981**
5	0.687	2.749	68.731	-	-	-	-	-	-
6	0.621	2.484	71.216	-	-	-	-	-	-
7	0.554	2.215	73.431	-	-	-	-	-	-
8	0.528	2.112	75.543	-	-	-	-	-	-
9	0.511	2.045	77.588	-	-	-	-	-	-
10	0.48	1.921	79.509	-	-	-	-	-	-
11	0.463	1.852	81.361	-	-	-	-	-	-
12	0.428	1.713	83.074	-	-	-	-	-	-
13	0.417	1.669	84.743	-	-	-	-	-	-
14	0.403	1.613	86.355	-	-	-	-	-	-
15	0.385	1.539	87.894	-	-	-	-	-	-
16	0.374	1.496	89.39	-	-	-	-	-	-
17	0.357	1.426	90.817	-	-	-	-	-	-
18	0.33	1.321	92.138	-	-	-	-	-	-
19	0.329	1.317	93.455	-	-	-	-	-	-
20	0.316	1.265	94.719	-	-	-	-	-	-
21	0.297	1.19	95.909	-	-	-	-	-	-
22	0.283	1.13	97.039	-	-	-	-	-	-
23	0.262	1.049	98.088	-	-	-	-	-	-
24	0.241	0.964	99.052	-	-	-	-	-	-
25	0.237	0.948	100	-	-	-	-	-	-

Table S5.5 Rotated Component Matrix for the post-00s cohort

Component	Factor Loadings				Communalities
	G1	G2	G3	G4	
1	0.194	0.429	0.226	0.531	0.555
2	-0.034	0.677	0.232	0.424	0.693
3	0.351	0.313	0.281	0.578	0.634
4	0.305	0.228	0.256	0.669	0.659
5	0.729	0.031	0.083	0.363	0.67
6	0.148	0.691	0.191	0.378	0.678
7	0.318	0.64	0.321	0.201	0.653
8	0.173	0.818	0.123	0.171	0.744
9	0.493	0.363	0.348	0.23	0.549
10	0.72	0.265	0.183	0.136	0.641
11	0.747	0.32	0.157	0.09	0.693
12	0.659	0.272	0.316	0.133	0.626
13	0.745	-0.034	0.303	0.167	0.676
14	0.428	0.632	0.26	0.052	0.652
15	0.386	0.479	0.465	0.121	0.609
16	0.215	0.573	0.547	0.15	0.696
17	0.356	0.285	0.627	0.257	0.667
18	0.286	0.241	0.69	0.268	0.689
19	0.24	0.269	0.759	0.202	0.747
20	0.252	0.259	0.735	0.201	0.711
21	0.397	0.174	0.652	0.184	0.647
22	0.703	0.033	0.328	0.098	0.613
23	0.343	0.451	0.536	0.015	0.608
24	0.546	0.411	0.462	0.019	0.681
25	-0.009	0.678	0.488	0.076	0.703

Rotation method: Varimax

Table S5.6 Cronbach's alpha coefficients of four latent variables for the post-00s cohort

	Cronbach's alpha	N of Items
G1	0.882	6
G2	0.865	5
G3	0.896	5
G4	0.686	2

Supplementary Table S6. Confirmatory factor analysis (CFA) and some statistics for the post-90s cohort IBM SPSS, IBM AMOS and Microsoft Excel.

Tabel S6.1 Model fit summary for the post-90s cohort

Model	CMIN/DF	GFI	NFI Delta1	IFI Delta2	TLI rho2	CFI	RMSEA
Default model							
Saturated model		1.000	1.000	1.000		1.000	
Independence model			.000	.000	.000	.000	

In Tabel S6.1, GFI = 0.931, NFI = 0.932, IFI = 0.955, TLI = 0.926, CFI = 0.954 (> 0.90 for all of them), CMIN/DF = 2.844 < 3, and RMSEA = 0.072 < 0.08, they fulfill the recommended cut-off values.

Tabel S6.2 Standardized Regression Weights for the post-90s cohort

			Estimate
L9	←	F1	0.756
L12	←	F1	0.795
L19	←	F1	0.715
L20	←	F1	0.649
L21	←	F1	0.657
L13	←	F2	0.765
L14	←	F2	0.578
L17	←	F2	0.883
L18	←	F2	0.802
L2	←	F3	0.715
L3	←	F3	0.752
L6	←	F3	0.713
L8	←	F4	0.69
L10	←	F4	0.681
L11	←	F4	0.708
L16	←	F4	0.753

Tabel S6.3 The results of CR and AVE for the post-90s cohort

Latent Variable	Item	Factor Loading	Squared Multiple Correlation (SMC)	Standardized Residual (1-SMC)	CR	AVE
F1	v1	0.756	0.572	0.428	0.84	0.514
	v2	0.795	0.632	0.368		
	v3	0.715	0.511	0.489		
	v4	0.649	0.421	0.579		
	v5	0.657	0.432	0.568		
F2	v1	0.765	0.585	0.415	0.847	0.586
	v2	0.578	0.334	0.666		
	v3	0.883	0.780	0.220		
	v4	0.802	0.643	0.357		
F3	v1	0.715	0.511	0.489	0.771	0.528
	v2	0.752	0.566	0.434		
	v3	0.713	0.508	0.492		
F4	v1	0.69	0.476	0.524	0.801	0.502
	v2	0.681	0.464	0.536		
	v3	0.708	0.501	0.499		
	v4	0.753	0.567	0.433		

Supplementary Table S7. Confirmatory factor analysis (CFA) and some statistics for the post-00s cohort using SPSSAU

Tabel S7.1 Model fit summary for the post-00s cohort

Common Indicators	GFI	NFI	IFI	TLI	CFI	RMSEA	$\chi 2$/df
Criteria	>0.9	>0.9	>0.9	>0.9	>0.9	<0.10	<3
Value	0.916	0.929	0.935	0.923	0.935	0.074	10.047

For the post-00s, in Tabel S7.1, CFI = 0.935, TLI = 0.923, IFI = 0.935, GFI = 0.916, and RMSEA = 0.074, all of them meet the criteria. Although χ^2/df = 10.047 exceeded conventional threshold, this is expected in large samples (n = 1,672) due to the sensitivity of the chi-square statistic to sample size, and therefore does not necessarily indicate inadequate model fit. Moreover, as shown in the following Tabel S7.2 and Tabel S7.3, factor loadings (i.e., Std. Estimates) ranged from 0.694 to 0.832, CR values ranged from 0.688 to 0.897, and AVE values ranged from 0.524 to 0.636. These results indicate acceptable convergent validity across constructs.

Tabel S7.2 Factor Loading Coefficient for the post-00s cohort

Latent Variable	Measurement Item(abbrev.)	p	Std. Estimate	SMC
G1	Emergency response	-	0.703	0.495
G1	Foreign policy	0	0.701	0.492
G1	Military strength	0	0.745	0.555
G1	Environment	0	0.781	0.609
G1	Infrastructure	0	0.785	0.617
G1	Social harmony	0	0.757	0.573
G2	Income balance	-	0.714	0.51
G2	Life satisfaction	0	0.737	0.544
G2	Wages	0	0.771	0.595
G2	Gov efficiency	0	0.796	0.634
G2	Price stability	0	0.761	0.579
G3	Norms Integrity	-	0.8	0.64
G3	Media governance	0	0.75	0.563
G3	Cultural export	0	0.795	0.632
G3	IP respect	0	0.832	0.692
G3	Overseas behaviour	0	0.808	0.653
G4	Work ethic	-	0.694	0.482
G4	Innovation	0	0.753	0.567

In Tabel S7.2, to enhance interpretability, the item numbers were replaced with the item abbreviations, i.e., brief semantic labels of one to two words that accurately convey their meaning.

Tabel S7.3 Discriminant validity indices for the post-00s cohort

Latent Variable	AVE	CR	MSV	ASV
G1	0.557	0.883	0.6	0.525
G2	0.572	0.87	0.65	0.554
G3	0.636	0.897	0.657	0.616
G4	0.524	0.688	0.657	0.636

Tabel S7.4 The results of HTMT (Heterotrait-Monotrait Ratio)

Latent Variable	G1	G2	G3	G4
G1	-			
G2	0.615	-		
G3	0.764	0.788	-	
G4	0.777	0.804	0.815	-
